# Pet Therapy as a Nonpharmacological Treatment Option for Neurological Disorders: A Review of the Literature

**DOI:** 10.7759/cureus.16167

**Published:** 2021-07-04

**Authors:** Catherine M Boldig, Nitin Butala

**Affiliations:** 1 Neurology, University of South Florida Morsani College of Medicine, Tampa, USA; 2 Neurology, Baptist Medical Center Jacksonville, Jacksonville, USA

**Keywords:** animal-assisted therapy, therapy dog, dog ownership, neurological disorders, endurance exercise

## Abstract

Animal therapy and ownership have been studied as a nonpharmacologic treatment option for cardiovascular and psychological disorders. Animal companionship is less studied in neurological disorders such as stroke, dementia, Parkinson’s disease, multiple sclerosis, Huntington’s disease, epilepsy, and acute brain injury. This review examines the effects that emotional support dogs, dog therapy, or dog ownership has on these specific neurological disorders. It may serve as a nonpharmaceutical option to improve patient symptoms, quality of life, or the disease course itself. Articles were gathered which studied the effect of animal-assisted therapy, pet therapy, dog ownership, and physical activity on neurological disorders. Studies relating to the topic were then assessed for the impact on neurological disorders which ranged from cognition, mobility, quality of life, mood, and improvement of disease course. Dog therapy and ownership were found to improve mood, quality of life, and disease symptoms across multiple neurological disorders. It also encouraged physical activity which was shown to help many diseases studied, even ones associated with skeletal muscle apoptosis, such as Huntington’s disease. Dog therapy and ownership are a safe and effective nonpharmaceutical approach to treating chronic and progressive neurological disorders.

## Introduction and background

Many individuals enjoy the companionship of a dog and may, unknowingly, incur a health benefit from ownership. Dogs have been studied for cardiovascular disease and have provided life-saving benefits. Studies have shown a 31% risk reduction of cardiovascular mortality and a 24% risk reduction of all-cause mortality when comparing dog owners to non-dog owners [1,2]. According to the American Heart Association, dog owners live longer and recover from major life illnesses, such as heart attack and stroke, quicker than non-dog owner counterparts [3]. This positive impact on life expectancy may occur because of a decrease in blood pressure, heart rate, lipid levels, and blood glucose levels [1]. The health benefits are also thought to be associated with a reduction of stress and increased physical activity because of dog walking [1]. An improvement in these variables would demonstrate that companionship with a dog would improve cardiovascular and neurological health. This could result in a reduced risk of stroke and other neurological disorders. Articles were included in the review if they used dogs amongst animals studied. Studies were excluded if they did not use dogs. Research by Muñoz et al. has summarized effects on cerebral palsy, autism, spasticity-related disease, dementia, and intellectual disability using horses, donkeys, fish, and dogs during therapy [4]. Motor, language, social interaction, and emotions improved with the use of support animals for these neurological disorders [4]. Having validity behind the use of a variety of animals, this paper focuses on dog ownership and dog-assisted therapy to determine if benefits to neurological diseases still exist. Since research is limited correlating the effects of dogs and neurological disorders, inclusion criteria were expanded to include studies with the effects of endurance exercise on disease course. These studies were included because dog ownership often correlates with increased walking.

## Review

Effect on stroke

Dogs may positively impact patients that suffered an ischemic or hemorrhagic stroke. This is demonstrated in the fact that stroke survivors that owned a dog had a 27% decreased risk of mortality compared to those who did not own a dog [3]. The neurovascular system is intimately related to the cardiovascular system and improved cardiovascular parameters may also represent measures of improved neurovascular health. These measurements consisted of improved blood pressure, heart rate, lipid levels, and blood glucose following dog walking [1]. Psychological improvements that were found consisted of improved independence and self-sufficiency in stroke patients that used dog therapy [5]. Dogs also provide a calming rehabilitation environment facilitating improved language and speech in patients with post-stroke aphasia [6]. Stroke rehabilitation can be a long and challenging process, and pet therapy can improve outcomes by providing companionship, encouragement, and motivation for physical activity.

Effect on Parkinson's disease

Dogs have also been shown to be an effective nonpharmaceutical treatment of Parkinson’s disease. Ownership of a dog has decreased the number of medications needed and improved the physical symptoms of those with early diagnosis of Parkinson’s disease [7]. The physical activity that comes with dog ownership improves fatigue, bowel function, and appetite [7]. Dogs encourage owners or therapy participants, to increase exercise by going on walks and increase activity by taking care of their daily needs. This is valuable for slowing the progression of the disease because physical activity has been shown to also improve cognitive function in Parkinson’s disease [8]. Specifically, over 24 months, three 60-minute exercises a week improved processing speed, attention, and mental flexibility [8]. Along with physical and cognitive improvements that walking a dog may provide Parkinson’s patients, being responsible for the care of a dog provides psychological benefits as well. These are important strides for patients who may have run out of treatment options. The Parkinson’s Foundation has created a program that offers service dogs to Parkinson’s patients. The dogs assist the patients with freezing episodes and pull them to help get moving [9]. Some dogs are trained to respond to commands and become rigid to help assist the patient get up when they have fallen [9]. They also provide confidence to move around safely and without injury [9]. Service dogs for Parkinson’s disease can help patients overcome both physical and psychological aspects of the disease.

Effect on multiple sclerosis

Previously, exercise was thought to be detrimental to those suffering from multiple sclerosis. Newer research has found a reduction in the number of relapses of disease [10]. These positive effects on the disease course may have concrete effects on the structure of the brain. The relapsing and remitting course of multiple sclerosis causes a reduction in the white and gray matter of the brain, including the cortex and deep brain structures [11]. Physical activity has been studied to reverse the effects of multiple sclerosis, increasing the size of the white matter, gray matter, and deep brain structures such as the hippocampus, thalamus, caudate, putamen, and pallidum [11]. A statistically significant increase in the size of these structures, measured with MRI, was found when subjects performed moderate to vigorous physical activity [11]. Activities could include a brisk walk with a dog. The National MS Society website offers assistance dogs for MS patients that are meant to help with mobility and keep the patient moving [12]. An assistance dog can also help guide and improve safety by alerting to sounds, helping with doors, pulling wheelchairs, assisting with balance, and turning light switches on and off [12]. The dogs help patients with activities of daily living in hopes that patients can stay active and independent.

Effect on dementia

Dementia is a progressive disease that causes impairment in thinking, memory, and personality which consequently affects activities of daily living. There is no curative treatment for dementia, however, dog-assisted therapy has been studied as a potential nonpharmaceutical treatment of disease. Patients with dementia exposed to animals appeared less depressed compared with those not exposed to animal-assisted therapy [13,14]. A reduction in agitation, anxiety, and increased episodes of smiling has also been noted [15]. Increased physical activity and at least temporary cognitive improvement have also been concluded when a dog is used during therapy with these patients [15]. This systematic review with a primary endpoint of the study being quality of life found improvement in health and happiness of patients suffering from dementia. Improvement in social and cognitive function occurs in dementia because dog ownership and therapy provide a purpose for these patients who often feel hopeless.

Effect on Huntington's disease

Huntington’s disease is a neurodegenerative disorder where pet therapy may be a nonpharmaceutical treatment option to improve the disease course and patient outlook. Mueller et al. described an improvement or decreased progression in components such as safety, motor function, cognition, body composition, quality of life, cardiovascular and mitochondrial function [16]. Skeletal muscle mitochondrial dysfunction is a common manifestation of Huntington’s disease [16]. An increase in mitochondrial content and capillary-to-muscle fiber ratio is noted to increase during physical activity such as walking or running [16]. The rapidly debilitating nature of Huntington’s disease is often traumatic but dog ownership would be one way to encourage endurance training and improve motor function.

Effect on acute brain injury

Animal-assisted therapy has also been used in patients with acute brain injury. Along with the previously mentioned neurological conditions, social isolation and loneliness are common problems reported by those with an acute brain injury [17]. Dogs have been reported as being calming for patients and provide a topic of conversation to those coping with social interaction difficulties [17]. The therapy dogs are also thought to help motivate the participant to work harder during therapy sessions [17]. Through improved motivation, social interactions, and integration into society, animal-assisted therapy helps these patients with a more seamless post-injury recovery.

Effect on seizures

Seizure dogs are service dogs trained specifically for patients with epilepsy and have been shown to provide great benefit. Their training allows them to assist by activating alarms, alerting caregivers of a seizure, and protecting patients from unsafe environments during an event [18]. Exercise has been shown to reduce seizure frequency in people suffering from epilepsy [19]. Initially, it was thought that stress from exercise could trigger seizures [19]. However, when studied, patients that exercised until exhaustion did not experience a seizure [19]. Exercise is also associated with lower rates of depression in epilepsy [19,20]. Dog ownership or therapy is a valuable treatment option for patients with epilepsy, who often have multiple medications that can cause drug interactions.

The benefit of dogs

The benefits associated with mental and physical health correlated with dogs specifically. Many of the benefits relating to dogs are acquired through physical activity that comes along with dog ownership and therapy. Dogs are usually the only pets that are walked daily. People tend to walk longer distances with their dogs than if they went on a walk by themselves [21]. Dog owners are more likely to engage in physical activity during their free time or make physical activity a priority [21]. Humans create a bond with dogs and find comfort in their presence. Dogs are a nonpharmaceutical option that has been shown to help treat diseases that have been difficult to clinically manage with medications [21]. Although all pets may cause improvement in mood and emotion, dog ownership and therapies have morbidity and mortality benefits, proven beyond those associated with other pets.

## Conclusions

Emotional support animals, especially dogs, are a popular companion that provides assistance to owners who may experience mental and physical challenges. This sparked the initial interest in how they affect those suffering from neurological disorders. There is research that studied the benefits dogs have on cardiovascular morbidity and mortality. We were interested in determining if this benefit also translated to neurovascular and neurological morbidity and mortality. Stroke survivors that are dog owners are shown to have a decrease in mortality compared to non-dog owners. Increased physical activity associated with dog ownership slows disease progression in Parkinson’s disease, increases the size of vital brain structures that deteriorate and cause disability in multiple sclerosis, and reduces seizure frequency in epilepsy. It also improves motor function in patients with Huntington’s disease. Dogs are associated with decreased agitation and thought to cause at least temporary cognitive improvement in patients with dementia. Dogs also help improve social interaction in those suffering from acute brain injury. Although the outcome of current studies has provided a promising benchmark, future studies demonstrating the effect of dog-assisted therapy on neurological disorders can progress knowledge on this topic. This review suggests that dog therapy and ownership is a safe and effective non-pharmaceutical option that may improve symptoms of chronic and progressive neurological conditions, patient psychology, and enhance well-being.
